# Suitable intravenous fluid for preventing dysnatremia in children with gastroenteritis; a randomized clinical trial

**DOI:** 10.15171/jrip.2016.15

**Published:** 2016-04-22

**Authors:** Kioomars Golshekan, Hamidreza Badeli, Mahboube Miri, Maryam Mirzaie, Afagh Hassanzadeh Rad, Fatemeh Salamat, Sepideh Abdi Tazeabadi, Nahid Bidar, Kobra Blouki-Moghaddam, Houman Hashemian

**Affiliations:** ^1^Pediatrics Growth Disorders Research Center, 17 Shahrivar Hospital, School of Medicine, Guilan University of Medical Sciences, Rasht, Iran; ^2^Chancellorship for Research, Guilan University of Medical Sciences, Rasht, Iran

**Keywords:** Gastroenteritis, Hyponatremia, Hypernatremia, Children

## Abstract

**Introduction:** Gastroenteritis (GE) is one of the most common pediatric diseases. Hyponatremia commonly occurs by administering hypotonic fluids to GE and hospitalized children. Yet, there is no consensus on the ideal method of treatment.

**Objectives:** we aimed to assess suitable intravenous (IV) fluid for preventing dysnatremia in children with GE.

**Patients and Methods:** This is a double blind randomized clinical trial, which was conducted on infants of 6 months up to 14 years children with GE. Children were randomly assigned in 2 different groups. Group A; received 20 cc/kg 0.9% isotonic saline as a bolus, and 0.45% hypotonic saline as sum of maintenance fluid and volume deficit. Group B was treated with the same bolus and 0.9% isotonic saline with 20 mEq/L KCl as sum of maintenance fluid and volume deficit. Blood and urine samples were taken at admission, 4 and 24 hours. Data were analyzed by independent t test, Mann-Whitney U test, Friedmann test, chi-square and 2-tailed repeated measurements by SPSS version 19.

**Results:** Baseline hyponatremia and isonatremia were detected in 24 (31.5%) and 51 (67.1%) patients, respectively. Mean level of sodium at T0, T4 and T 24 mentioned no significant difference between groups. No hypernatremia was noted by administering isotonic saline. Results showed that 4 and 24 hours after administration isotonic saline, the mean plasma sodium differed significantly in baseline hyponatremic patients. However, no significant difference was noted after 4 and 24 hours in group A.

**Conclusion:** According to the considerable effect of isotonic saline on hyponatremic patients, it seems that administering isotonic fluids regardless of the types of dysnatremia can be recommended to lessen clinicians’ conflicting decision-making in selecting an appropriate fluid.

**Implication for health policy/practice/research/medical education:**

According to the considerable effect of isotonic saline on hyponatremic patients, it seems that administering isotonic fluids regardless of the types of dysnatremia can be recommended to lessen clinicians’ conflicting decision making in selecting an appropriate fluid at the commence of treatment in patients with gastroenteritis (GE).

## Introduction

Gastroenteritis (GE) is one of the most common pediatric diseases and dehydration induced by it can cause mortality and morbidity in developing countries (1). Although, oral rehydration therapy (ORT) is the first step for treating patients. However, intravenous (IV) fluids should be indicated when oral therapy cannot be tolerated (2). Administering IV hypotonic saline as maintenance and fluid loss is still one of the most common methods of GE treatment (3).

But, investigations mentioned that hyponatremia commonly occurs by administering hypotonic fluids to GE (4) and hospitalized children (5,6). Hyponatremia (plasma sodium less than 135 mmol/L) has been known as a cause of morbidity and mortality in hospitalized children (7). It can induce cerebral hernia, convulsion, respiratory arrest, pulmonary edema and irreversible neurologic sequelae (8). The release of antidiuretic hormone (ADH) as a result of non-osmotic stimulus such as nausea, vomiting, ache, tension, trauma, and opioids is the main common cause of hyponatremia in hospitalized children (9). Therefore, investigators declared using isotonic saline as the routine maintenance solution for GE patients (10-13).

As, there is no consensus on the ideal method of treatment, we aimed to assess suitable IV fluid for preventing dysnatremia in children with GE.

## Patients and Methods

**Figure 1:**
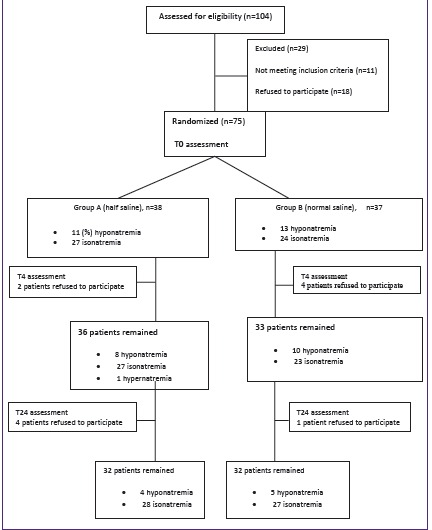
Enrollment and outcomes.

This is a double blind randomized clinical trial, which was conducted on 6 months infants up to 14 years children admitted in 17 Shahrivar Children’s hospital in Rasht, north of Iran during January 2013 to April 2014. Patients with GE and inability to tolerate ORT, lack of any known confounding diseases such as abnormality of ADH secretion, pituitary or hypothalamic diseases, renal diseases, acute or chronic lung disease and no previous and/or recent usage of ADH stimulating secretion drugs were included to the study. During study protocol, patients with loss of consciousness, toxicity and anemia were excluded. Informed consents were obtained from parents and children were randomly assigned by blocking in two different groups and matched based on degree of dehydration. Group A received 20 cc/kg 0.9% isotonic saline solution as a bolus as needed and 5% dextrose in hypotonic saline solution (0.45%) with 20 mEq/L KCL as sum of maintenance fluid and volume deficit. Group B was treated with 20 cc/kg 0.9% isotonic saline solution as a bolus as needed and 5% dextrose in isotonic saline solution (0.9%) with 20 mEq/L KCL as sum of maintenance fluid and volume deficit (Figure 1).

The daily water requirements for treatment indicated based on weight and the slow replacement protocol was used for infusion. Blood samples were taken at admission (T0), 4 (T4) and 24 (T24) hours after commencing IV therapy for measuring levels of sodium, potassium, BUN, creatinine, PH, PCO2 and HCO3. Urine samples were also collected to determine sodium, potassium, creatinine and specific gravity concomitantly with blood samples.

### Ethical issues

The research followed the tenets of the Declaration of Helsinki. Informed consents were obtained from parents and the research was approved by the Ethics Committee of Guilan University of Medical Sciences. This clinical trial had a registration ID of IRCT NO: IRCT201211261545N2 in Iran (http://en.search.irct.ir/view/11579).

### Statistical analysis

Data were analyzed by independent *t* test, Mann-Whitney U test, Friedmann test, chi-square and two tailed repeated measurements in the statistical package for social sciences (SPSS Inc, Chicago, version 19.0 for Windows). Statistical significance considered by *P* value less than 0.05 and 95% CI was noted.

## Results

This study comprised of 75 patients. Mean age and weight were 36±3.1 months and 12.9±10.6 kg, respectively. The majority of patients (60%) were male. Results showed no significant difference between groups regarding age, sex and weight (*P*>0.05). All patients had mild to moderate degree of dehydration. Baseline hyponatremia and isonatremia were detected in 24 (31.5%) and 51 patients (67.1%), respectively.

Table 1 and 2 demonstrate serum and urine parameters. Repeated measurement analysis of variance (ANOVA) noted no significant difference in groups A (*P*=0.8) and B (*P*=0.49) regarding mean sodium of T0, T4 and T24. Also, mean sodium between groups based on T0, T4 and T24 mentioned no significant difference (*P*=0.97). Serum parameters in 2 groups were presented in Table 2.

**Table 1:** Serum parameters in two groups at different times

	**Group A (hypotonic saline)**			** Group B (isotonic saline)**		
	** T0 **	** T4 **	**T24**	** T0**	** T4**	** T24**
Mean sodium	136.47 ± 4.42	136 ± 4.7	136.67±2.3	136.23 ± 3.64	136.39 ± 3.4	136.94±2.75
Mean potassium	3.9 ± 0.61	4 ± 0.57	3.91±0.48	4 ± 0.49	3.9 ± 0.44	3.99±0.51
Mean BUN	12.19 ± 4.4	9.63 ± 3.4	7.37±1.97	11.74 ± 5.1	8.9 ± 3.1	7.17±1.28
Mean creatinin	0.59 ± 0.01	0.55 ± 0.09	0.53±0.08	0.57 ± 0.08	0.55 ± 0.08	0.54±0.08
Mean PCO2	31.71 ± 5.52	32.74 ± 5.38	33.96±5.74	30.84 ± 5.16	31.80 ± 4.35	32.86±4.09
Mean Hco3	16.99 ± 3.39	17.47 ± 3.3	18.99±3.6	16.95 ± 3.66	17.49 ± 2.83	18.95±2.6
Mean pH	7.33	7.34	7.36	7.34	7.35	7.36

Abbreviations: BUN, blood urea nitrogen.

**Table 2:** Urine parameters in groups

	**Group A (hypotonic saline)**			** Group B (isotonic saline)**		
	** T0 **	** T4 **	**T24**	** T0**	** T4**	** T24**
Mean sodium	64.59 ± 42.75	88.12 ± 59.25	85.41 ± 39.31	94.05 ± 59.66	119.22 ± 69.61	165.67 ± 164.79
Mean potassium	29.16 ± 33.50	21.48 ± 18.95	20.72 ± 30.52	31.4 ± 24.81	22.43 ± 19.80	15.19 ± 9.26
Mean creatinine	61.56 ± 154.39	37.87 ± 35.59	21.24 ± 22.93	36.51 ± 28.89	25.80 ± 20.73	23.09 ± 16.72
Specific gravity	1.016 ± 0.009	1.016 ± 0.009	1.010 ± 0.005	0.990 ± 0.167	0.987 ± 0.169	0.994 ± 0.13

At T0, hyponatremia was noted in 28.9% of group A which reduced to 22.2% at T4 and 12.5% at T24. Results indicated no significant difference in intra-individual times in this group (*P*=0.08). However, 35.1% of participants in group B noted hyponatremia at T0 which reduced to 30.3% at T4 and 15.6% at T24. Friedmann test demonstrated significant difference in intra-individual times in group B (*P*=0.05). No hypernatremia was noted by administering isotonic saline solution (Table 3).

**Table 3:** Dodium status in groups

	**Group A (hypotonic saline)**			** Group B (isotonic saline)**		
	** T0 **	** T4 **	**T24**	** T0**	** T4**	** T24**
Serum sodium status, n (%)						
Hyponatremia	11 (28.9)	8 (22.2)	4 (12.5)	13 (35.1)	10 (30.3)	5 (15.6)
Isonatremia	27 (71.1)	27 (75)	28 (87.5)	24 (64.9)	23 (69.7)	27 (84.4)
hypernatremia	0 (0)	1 (2.8)	0 (0)	0 (0)	0 (0)	0 (0)
Total, n (%)	38 (100)	36 (100)	32 (100)	37 (100)	33 (100)	32 (100)

Our results showed that after 4 and 24 hours administering isotonic saline solution, the mean plasma sodium differs significantly in baseline hyponatremic group (respectively, *P*=0.025 and *P*=0.000) , however, no significant difference was noted 4 and 24 hours after administering hypotonic saline solution (*P*>0.05).

Mann-Whitney U test obtained no significant difference in T0, T4 and T24 regarding sodium status (*P*=0.56, p=0.45, *P*=0.72, respectively).

## Discussion

Substitution of fluids and electrolytes loss is the main method of treatment in patients with GE. Although, ORT is the first step for treating patients with GE; but IV fluids should be indicated when oral therapy cannot be tolerated (2).

For about 50 years, pediatricians commonly recommended the Holliday and Segar guidelines for administering “maintenance” (IV) fluids (14). However, investigators demonstrated that hypotonic fluid might cause hyponatremia, which causes morbidity and death in hospitalized children (7). Therefore, according to previous investigations, administering isotonic saline solution in children admitted to emergency departments had been proposed to eliminate iatrogenic hyponatremia. Yet, there is no consensus on the ideal method of treatment (10-13).

Our results indicated administering isotonic saline solution decreased hyponatremia statistically significant which was similar with previous investigations (5,6). Furthermore, 4 and 24 hours after administering isotonic saline solution, the mean plasma sodium differs significantly in baseline hyponatremic group (*P*=0.025 and *P*=0.000, respectively) nonetheless, no significant difference was noted after 4 and 24 hours after administering hypotonic saline solution (*P*>0.05).

Results showed the preventive effects of isotonic saline solution in increasing plasma sodium concentration in hyponatremic (high rate of hyponatremia at the admission time) and isonatremic patients, it seems that administering isotonic saline solution might be a suitable fluid regardless of the types of dysnatremia. It can lessen clinicians’ conflicting decision-making in selecting an appropriate fluid at the commence of treatment in patients with GE.

Also, no hypernatremia was mentioned in isotonic saline group which was similar with previous results (15,16).

Our results noted no significant difference between groups regarding mean plasma sodium during periods, which was similar with the results mentioned by recent articles (15,17). Whereas, Sánchez-Bayle et al noted the use of hypotonic fluid did not increase the risk of hyponatremia in GE patients with mild to moderate dehydration (15). In addition, Saba et al noted no significant difference regarding the rate of change and absolute change in serum [Na] between children with medical illnesses admitted by the emergency department (medical), and children admitted following elective surgery (surgical) (17). However, previous randomized trials noted significantly greater drops in plasma sodium by administering hypotonic solutions compared with isotonic ones (18-22).

The discrepancy between the results of studies mentioned above firstly may be due to the percentage of patients’ dehydration status. While in our study and the study by Sánchez-Bayle et al, patients in both groups had mild to moderate degrees of dehydration which could not stimulate volume dependant ADH secretion (15) and secondly, it may be due to less nonosmotic stimuli of ADH secretion in GE than surgery and critical care wards.

## Conclusion

According to the considerable effect of isotonic saline on hyponatremic patients, it seems that administering isotonic fluids regardless of the types of dysnatremia can be recommended to lessen clinicians’ conflicting decision making in selecting an appropriate fluid at the commence of treatment in patients with GE.

## Limitations of the study

Although, results mentioned significant effect of isotonic saline on hyponatremic patients, it seems that further investigations on more patients can offer new insights to treat patients with GE.

## Authors’ contribution

KG and HB conceptualized and designed the study, drafted the initial manuscript, reviewed and revised the manuscript. MahM and MarM collected data and revised the manuscript. AHR coordinated and supervised data collection, drafted the initial manuscript and revised the manuscript and critically reviewed the manuscript. FS assessed the statistical analysis and critically reviewed the manuscript. SAT, NBM and HH drafted the initial manuscript and reviewed and revised the manuscript. All authors approved the final manuscript as submitted and agree to be accountable for all aspects of the work.

## Conflicts of interest

The authors have indicated they have no potential conflicts of interest to disclose.

## Ethical considerations

Ethical issues (including plagiarism, data fabrication, double publication) have been completely observed by the authors.

## Funding/Support

This study was conducted with financial support provided by vice chancellor of research in Guilan University of Medical Sciences.

## References

[1] Das JK, Salam RA, Bhutta ZA (2014). Global burden of childhood diarrhea and interventions. Curr Opin Infect Dis.

[2] Ciccarelli S, Stolfi I, Caramia G (2013). Management strategies in the treatment of neonatal and pediatric gastroenteritis. Infect Drug Resist.

[3] Carcillo JA (2014). Intravenous fluid choices in critically ill children. Curr Opin Crit Care.

[4] Hanna M, Saberi MS (2010). Incidence of hyponatremia in children with gastroenteritis treated with hypotonic intravenous fluids. Pediatr Nephrol.

[5] Hoorn EJ, Geary D, Robb M, Halperin ML, Bohn D (2004). Acute hyponatremia related to intravenous fluid administration in hospitalized children: an observational study. Pediatrics.

[6] Au AK, Ray PE, McBryde KD, Newman KD, Weinstein SL, Bell MJ (2008). Incidence of postoperative hyponatremia and complications in critically-Ill children treated with hypotonic and normotonic solutions. J Pediatr.

[7] Auroy Y, Benhamou D, Péquignot F, Jougla E, Lienhart A (2008). Hyponatraemia-related death after paediatric surgery still exists in France. Br J Anaesth.

[8] Sterns RH, Nigwekar SU, Hix JK, eds eds (2009). The treatment of hyponatremia. Semin Nephrol.

[9] Koczmara C, Wade A, Skippen P, Campigotto M, Streitenberger K, Carr R (2009). Hospital-acquired acute hyponatremia and reports of pediatric deaths. Dynamics.

[10] Moritz ML, Ayus JC (2003). Prevention of hospital-acquired hyponatremia: a case for using isotonic saline. Pediatrics.

[11] Moritz ML, Ayus JC (2005). Preventing neurological complications from dysnatremias in children. Pediatr Nephrol.

[12] Moritz ML, Ayus JC (2004). Hospital-acquired hyponatremia: why are there still deaths?. Pediatrics.

[13] Moritz ML, Ayus JC (2008). 09% saline solution for the prevention of hospital-acquired hyponatremia: Why is there still doubt?. J Pediatr.

[14] Holliday MA, Segar WE (1957). The maintenance need for water in parenteral fluid therapy. Pediatrics.

[15] Sánchez-Bayle M, Martín RM, Fernández JC, Pinto EV (2014). Fluid therapy and iatrogenic hyponatraemia risk in children hospitalised with acute gastroenteritis: prospective study. Nefrologia.

[16] Choong K, Kho ME, Menon K, Bohn D (2006). Hypotonic versus isotonic saline in hospitalised children: a systematic review. Arch Dis Child.

[17] Saba TG, Fairbairn J, Houghton F, Laforte D, Foster BJ (2011). A randomized controlled trial of isotonic versus hypotonic maintenance intravenous fluids in hospitalized children. BMC Pediatr.

[18] Neville KA, Sandeman DJ, Rubinstein A, Henry GM, McGlynn M, Walker JL (2010). Prevention of hyponatremia during maintenance intravenous fluid administration: a prospective randomized study of fluid type versus fluid rate. J Pediatr.

[19] Yung M, Keeley S (2009). Randomised controlled trial of intravenous maintenance fluids. J Paediatr Child Health.

[20] Neville KA, Verge CF, Rosenberg AR, O’Meara MW, Walker JL (2006). Isotonic is better than hypotonic saline for intravenous rehydration of children with gastroenteritis: a prospective randomised study. Arch Dis Child.

[21] Montanana PA, Alapont VM, Ocon AP, Lopez PO, Prats JLL, Parreño JD (2008). The use of isotonic fluid as maintenance therapy prevents iatrogenic hyponatremia in pediatrics: A randomized, controlled open study. Pediatr Crit Care Med.

[22] Kannan L, Lodha R, Vivekanandhan S, Bagga A, Kabra SK, Kabra M (2010). Intravenous fluid regimen and hyponatraemia among children: a randomized controlled trial. Pediatr Nephrol.

